# Influence of social experiences in shaping perceptions of the Ebola virus among African residents of Hong Kong during the 2014 outbreak: a qualitative study

**DOI:** 10.1186/s12939-015-0223-6

**Published:** 2015-10-05

**Authors:** Judy Yuen-man Siu

**Affiliations:** David C. Lam Institute for East–West Studies (Environment, Health, and Sustainability working group), Hong Kong Baptist University, Kowloon Tong, Hong Kong

**Keywords:** Ebola virus disease (EVD), Social experiences, Stigma, Perceptions, African, Ethnic minorities, Hong Kong

## Abstract

**Introduction:**

The outbreak of Ebola virus disease (EVD) in Africa in 2014 attracted worldwide attention. Because of the high mortality rate, marginalised social groups are vulnerable to disease-associated stigmatisation and discrimination, according to the literature. In Hong Kong, ethnic minorities such as Africans are often disadvantaged groups because of their low position in the social hierarchy. In 2011, approximately 1700 Africans were residing in Hong Kong. Their overseas experiences during the EVD outbreak were not well documented. Therefore, this study investigated the EVD-associated stigmatisation experiences of African residents of Hong Kong with chronic illnesses, and how these experiences shaped their perceptions of EVD.

**Methods:**

A qualitative design with 30 in-depth semistructured interviews was conducted with chronically ill African residents of Hong Kong.

**Results:**

The interview data showed that the sampled Africans often experienced stigmatisation in their workplaces and in the community during the EVD outbreak. Their experiences of EVD-associated stigma were correlated to the embedded social and cultural values regarding ethnic minorities in Hong Kong. These experiences of being stigmatised shaped the perceptions of the Africans of EVD, leading them to view EVD as shameful and horrifying. They also perceived EVD as retribution and was introduced by Westerners. The participants’ perceptions of EVD influenced their responses to and behaviour towards EVD, which may have posed potential threats to Hong Kong’s public health.

**Conclusions:**

The EVD outbreak was not the only cause of the participants’ stigmatisation; rather, their EVD-associated experiences were a continuation and manifestation of the embedded social and cultural values regarding ethnic minorities in Hong Kong. The experiences of being stigmatised shaped the participants’ perceptions of EVD. Because of their marginalised social position and isolation from the main community, the participants had extremely limited access to reliable information about EVD. As a result, they used their own cultural beliefs to understand EVD, which might have ultimately influenced their health behaviours. The experiences of the participants showed that ethnic minorities in Hong Kong were in need of more culturally responsive social and health care support to obtain reliable information about the nature of and preventive measures against EVD.

## Introduction

The outbreak of Ebola virus disease (EVD) in 2014 attracted worldwide attention. The outbreak originated in West Africa and swept through numerous African countries [1]. Because EVD is a highly infectious disease, the increasing traffic and interaction between West Africa and other countries because of globalisation meant that EVD had high potential to be transmitted to outside Africa [2]. EVD first emerged in Africa in 1976, and it typically occurs in the tropical regions of Central and West Africa [3]. Since EVD emerged, it has appeared in different parts of Africa [2]. The disease has a high mortality rate, with an average fatality rate of 50 % generally; fatality rates varied from 25 to 90 % in past outbreaks [3]. There is no licensed treatment for EVD, and early supportive care is the only measure and treatment that improves survival [2].

Disadvantaged social groups such as ethnic minorities are documented as being highly vulnerable to contracting infectious diseases and experiencing associated stigmatisation and discrimination [4]. In Hong Kong, ethnic minorities comprise 4 % of the populations [5], and approximately 1700 Africans were residing in Hong Kong in 2011, according to government statistics [6]. These African residents were mostly from Algeria, Nigeria, Ghana, and Cameroon, and they mostly worked in importing and exporting [7]. Because the EVD outbreak originated in West Africa, African residents in Hong Kong experienced stigmatisation and discrimination following the EVD outbreak. However, there is scant literature concerning the experiences of the migrant Africans overseas during the EVD outbreak.

Infectious diseases do not merely endanger people’s health; they also manifest a country’s embedded social and cultural values. Infectious diseases such as HIV/AIDS and tuberculosis often demonstrate and manifest social inequalities in terms of race, social hierarchy, and gender [8]. Amongst these social inequalities, stigmatisation of and discrimination against ethnic minorities are common [8]. Take HIV/AIDS as an example; social minorities such as homosexuals and Blacks are often stigmatised as high-risk groups and thus discriminated against [9]. Migrant Africans have been documented as one of the ethnic minorities most vulnerable to disease-associated stigmatisation and discrimination [10]. Because of the low position in the social hierarchy, low education levels, and difficulty to obtain social resources of ethnic minorities, they are often deprived of receiving proper treatment when they become ill [8]. The lack of social awareness of the difficulties encountered by ethnic minorities also creates difficulty for these minority groups to access health care resources for avoiding infectious diseases [8]. When outbreaks occur, ethnic minorities are thus easily infected and are scapegoated as a result [8].

EVD is an infectious disease with high mortality that mostly emerges in Africa, and, according to the literature, marginalised social groups are more vulnerable to stigmatisation when new diseases emerge [4]. Therefore, migrant Africans—as marginalised ethnic minorities in most communities—are assumed to be highly vulnerable to stigmatisation and discrimination associated with EVD. In Hong Kong, ethnic minorities, such as Africans, are often marginalised because of their ethnicity and low position in the social hierarchy. In addition, this highly vulnerable social group has low social visibility. This stigmatised position can put these Africans in a disadvantaged position when an EVD outbreak occurs, leading to their possible higher morbidity, which could pose a risk for the entire community’s public health. Furthermore, stigmatisation can motivate patients to conceal their sickness [8, 11, 12], which is detrimental to the effective control of infectious diseases. Therefore, understanding the experiences of these migrant ethnic minorities during infectious disease outbreaks is crucial. However, there is a paucity of literature concerning the experiences of migrant Africans during the EVD outbreak. The social and cultural issues raised by EVD are not well documented, despite the fact that these issues can severely affect people’s perceptions of the disease and their corresponding health behaviours, and thus affect the public health of the entire community. Hence, this study was conducted to fill this gap in the literature by providing a case study on the EVD-associated stigmatisation experienced by African residents of Hong Kong, how this stigma can be explained by the embedded social and cultural values regarding ethnic minorities in Hong Kong, and how the stigma shaped the Africans’ perceptions of EVD, which in turn could have posed a risk to public health. Because many countries have become multicultural under the influence of globalisation, this article can guide health authorities in designing a culturally responsive infection control policy for these ethnic minority groups.

## Methods

In this study, a qualitative design using in-depth individual semistructured interviews was adopted. A total of 30 African residents of Hong Kong were recruited from Chungking Mansions in Hong Kong by purposive sampling. Chungking Mansions, located in Tsim Sha Tsui—a major commercial and tourist area in Hong Kong—is a 17-storey building with numerous low-priced guest houses, and attracts many Africans staying in the city for work and business. According to a recent ethnographic study, a large majority of the people working in the building are Africans [13], and they mostly work in the trade or catering sector [13].

### Participants

All 30 participants were ethnic Africans aged between 30 and 60 years. Most of the participants were in their 30s or 40s. Because this study examined the experiences of EVD-associated stigmatisation experienced by Africans in Hong Kong, and how their experiences were correlated with the embedded social and cultural values of Chinese Hong Kong people regarding ethnic minorities, only Africans who had already lived in Hong Kong for at least 7 years and had obtained residency were sampled. Some of the participants had lived in Hong Kong for more than 10 years. Such purposive sampling ensured that the participants had sufficient social experiences in Hong Kong. In addition, because the literature indicates that chronically ill patients are more vulnerable to disease-associated stigmatisation [14], people with chronic conditions were purposively sampled.

All the sampled participants were men, and all of them were single. The participants came to Hong Kong for work or business, and their family members were still residing in Africa. The participants came from different African countries, namely Nigeria, Algeria, Ghana, Cameroon, and Uganda. The participants came to Hong Kong because of the job recommendation from friends or relatives, and they commonly believed that making a higher income was easier in Hong Kong. All of the participants were employed in trade, sales and retail, or catering, except for one participant working in information technology. Most of the participants were working in Chungking Mansions, though a few of them were working nearby. To save on transportation, most of the participants lived in the same district as they worked, whereas a few of them were commuters and lived in rural area of Yuen Long.

The sampled participants had all received some form of education. One participant was a university graduate, 15 had entered secondary school, 13 had finished primary school, and one had entered but not completed primary school.

All of the participants reported at least one chronic condition. They mostly had hypertension or diabetes mellitus, and a few of them had chronic liver conditions and sickle cell anaemia. All of them were receiving following-up treatment to manage their chronic conditions in public hospitals.

### Data collection

Thirty men from Africa were identified and sampled for this study by process of purposive sampling in Chungking Mansions in Hong Kong. In-depth semistructured interviews were conducted with the participants in July and August 2014, during which time the first cases of EVD were confirmed overseas. The participants were approached and recruited when the researcher was conducting observational fieldwork in Chungking Mansions. Most of the participants were interviewed immediately after consenting to be interviewed, whereas seven participants scheduled interviews at a later, more convenient date and time.

Prior to the interviews, an interview question guide was developed. The guide was designed based on the literature about infectious diseases and ethnic minorities and the stigmatisation and discrimination of ethnic minorities, as well as on written and visual documentaries about ethnic minorities in Hong Kong. The question guide was used throughout the interview process to guide the discussion, and to ensure that the discussion stayed in relation to the research questions and followed a focused direction [15]. The guide contained a set of open-ended questions aimed to elicit participant experiences about being stigmatised in Hong Kong during the EVD outbreak, how these experiences were correlated with the embedded social and cultural values of Hong Kong, and how these experiences shaped their perceptions of EVD. The questions were as follows:

1. What is your impression on Ebola?
2. How do those in your social network (e.g., friends, colleagues, and family members) think about Ebola?
3. How do you respond to the Ebola outbreak?
4. Are there any differences in your experiences before and after the Ebola outbreak? If yes, can you talk about the difference that you have experienced?
5. Are there any differences in your experiences when you receive follow-up treatment for your chronic conditions before and after the Ebola outbreak? If yes, can you talk about the difference that you have experienced?
6. Do you think if there is any difference about how Hong Kong people view you before and after the Ebola outbreak?
7. How do you feel with these differences and changes about Hong Kong people’s view on you before and after the Ebola outbreak?
8. Do these experiences and changes affect you? If yes, what is the influence?
9. Can you talk more about your experience after you have lived in Hong Kong?
10. How do you think about Hong Kong people? From your experience, how do Hong Kong people view you?

The interviews were conducted with the participants on an individual basis. All of the interviews were conducted by the researcher to maintain consistency and ensure quality, and follow-up questions were asked during the interviews to collect more in-depth data from the participants. Interviews being conducted by a single researcher can minimise the risk of insufficient data collection that may be introduced by another interviewer. All the interviews were conducted in English, which was the common spoken language of the participants and researcher. The interviews were held in the workplaces of the participants for their convenience, and were audio-recorded with their consent. Each interview lasted 1–1.5 h. As an incentive, each participant was given a HK$100 supermarket cash coupon upon completion of the interview.

### Data analysis

Quick data analysis was conducted during the interviews to ascertain what was known and what topics needed to be explored further [16]. The interviews recordings were transcribed verbatim by two student assistants. The researcher then crosschecked the transcribed interviews with the recordings to ensure accuracy. A phenomenological analysis of the data was conducted by the researcher to understand the meanings of the participants’ experiences. Interview transcripts were analysed line by line, segmented into meaning units, and subsequently collapsed into categories. The major themes in the data were identified [17] through abstraction and constant comparison. A coding table was developed according to the inductive coding process, allowing the discovery of patterns of behaviours and thoughts [15]. The coding table identified themes, categories, and codes with supporting interview quotes. New thematic codes that emerged from the data were added to the coding table, and repetitive codes, categories, and themes were noted and highlighted to examine the frequency of their occurrence. Memos were used to record ideas and commentary during the coding process. The analytic procedures, codings, and findings were documented in the codebook to ensure the consistency and accuracy of the analysed data. Data saturation was achieved.

Direct interview quotations from the participants were included in the analysis so that their ideas were clearly represented and for the analysis to achieve credibility. Neutrality was established and the findings were grounded in the interview data rather than the researcher’s bias, motivation, or interest. Because this research was conducted by a single researcher, coding and recoding of the transcripts was performed to establish reliability and confirmability. The recoding (crosschecking) was performed 1 month after the initial coding to ensure that the analysis was clear and free of ambiguity and overlaps.

### Ethical considerations

The Committee on the Use of Human and Animal Subjects in Teaching and Research at Hong Kong Baptist University approved the study prior to the fieldwork. Participation in the study was voluntary. All the participants were provided with an information sheet in English explaining the purpose and nature of the study. The participants were also informed about the use of interview data, such as publication in academic journals with all personal identification removed. Verbal explanation and clarification was also offered to the participants before the interviews. Written consent was obtained from each participant to ensure that they were clear about the purpose of the interviews and use of data for academic publication. All the participants were assured of their freedom to withdraw from the study at any time. To protect the participants’ privacy, no participant identifiers were mentioned during the interviews. Each participant was represented by a code in the data and interview transcripts to further protect their privacy. All data and field notes were stored in locked files and treated with strict confidentiality. The audio recording of the interviews were destroyed after the interviews had been transcribed and checked.

## Results

The interview data showed that the participants commonly experienced EVD-associated stigmatisation in their workplaces and in the community following the EVD outbreak in West Africa. Stigmatisation of the participants became tangible and noticeable after the mass media in Hong Kong began widely reporting on the outbreak. Regarding the sources of stigmatisation of the participants, Chinese Hong Kong citizens were the key players, though stigmatisation from other ethnic minority groups was also common for the participants. The participants’ experiences of stigmatisation were not all due to the EVD outbreak; rather, their experiences were correlated with the embedded social and cultural values regarding ethnic minorities in Hong Kong. The experiences of the participants, in turn, shaped their perceptions of EVD and their corresponding health behaviour, which had crucial implications for the public health of ethnic minorities.

### Stigmatisation in the workplace

The workplace was a common site at which the participants experienced EVD-associated stigma. Most of the participants were working in the trade or catering business, and Chinese Hong Kong citizens were their main source of customers. During the outbreak, many participants noticed stigmatising behaviour by Chinese Hong Kong citizens. The following experience, as described by one participant, was common among the interviewees:

The business has dropped a lot. No Hong Kong people come in to eat now. Even though few people might stop by, they felt hesitated in touching our things. Sometimes I even heard that they said “Are they Africans? Do they have Ebola?” They are afraid of us for having Ebola. I once tried to ask some Hong Kong people to come in to eat, but they were gossiping softly, and then they left.

In addition to Chinese Hong Kong citizens, the colleagues of the participants were also players in the stigmatisation experiences. These colleagues were also ethnic minorities, and came from Pakistan, India, and Nepal. One participant recalled how he was stigmatised by his Pakistani colleagues:

Our relationship was not good when I first worked here because we are in different [ethnic] groups and we are not friend with each other. My wages is lower than them so they think I am competing with them. When the Ebola comes, they keep saying bad words on me, and blaming me for making the business worse. To protect their own sales performance, they even tell the customers that I am from the “country of Ebola”, which scares many customers off from me. I receive much lesser commission now, and my boss blames me now for making his business drops.

### Stigmatisation from the community

All the participants also experienced EVD-associated stigmatisation from the community. They often experienced stigmatisation when in and around Chungking Mansions, which is famous among Chinese Hong Kong citizens for ethnic minority businesses. One participant recalled the changes he observed in Chinese Hong Kong citizens’ attitudes towards Africans after the EVD outbreak:

I think many Hong Kong people are afraid of us. They do not go inside [Chungking Mansions] after the Ebola [outbreak]. Before the Ebola, many Hong Kong people would go inside for food. However, only a few Hong Kong people would go inside [the building] now. It is so quiet here. Many people keep a distance from us and from the building. Obviously they are scared if we, and the building, have Ebola. They seem to think that we all have Ebola.

Being stigmatised in public transport was also common for almost all the participants. One participant discussed the difference he noted when riding on buses before and after the outbreak:

After the Ebola [outbreak], no one wants to sit close to me. When I sat down, the people who have been sitting close to me immediately left their seats. They moved to other seats which were far away from me. No one took the seat next to me even though the bus was full. It just appeared that they were really afraid of me.

Several of the participants also experienced stigmatisation from neighbours:

My neighbours have started to avoid me since the Ebola [outbreak]. As I am living in a shared flat that I have to share the toilet and kitchen with other neighbours, they have become picky on my hygiene now. They think that I am dirty because I am a “black ghost” [a derogatory slang Cantonese Chinese term for Black people]. They keep telling me that I should keep the flat clean. The landlord even warns me that I should not go back to Africa these days, otherwise I have to leave [the flat if I return]. My neighbours have become monitors now, and they keep tracking if I am still coming back every day. I am sure they will report to the landlord if I do not come back for one day.

Because the participants had chronic conditions that required follow-up treatment at public hospitals, hospitals became another place at which they experienced stigmatisation. Stigmatisation from other patients was reported by more than half of the participants. One participant described how he was stigmatised while waiting for treatment:

After the Ebola [outbreak], many Hong Kong people are scared of me. No one wants to sit close to me while we are waiting [for treatment]. I remember at one time, several people with the same skin colour as mine were waiting, and all [the other] people left their seats and sat far away from us, though only I am an African. I really feel doubtful if they really know how to distinguish Africans; probably they just think that all people with black skin are from Africa. I also overheard their conversation [which was] about treating Ebola, and they stared at me unfriendly. I am sure they were gossiping about me.

A few of the participants mentioned that they experienced stigmatisation from health care providers while receiving follow-up treatment from public hospitals. One participant recalled how he was shocked by the nurses who treated him:

The nurses were afraid of me. They wore full protective gear, with facemask, face shield, gown, hat, and gloves, when having [preconsultation] examination on me. They kept asking me if I am from West Africa, and if I had been to West Africa in these few months. Also, they asked me several times if I had contacted any patients with Ebola. I was not sure if they were just more cautious about Ebola these days; but they did not wear the same set of things and ask the same set of questions when they were seeing other Hong Kong patients. This made me feeling very bad, and made me feeling I am very different and dirty, just because I am an African.

### Correlation between the EVD-associated stigma and embedded social and cultural values regarding ethnic minorities in Hong Kong

The EVD-associated stigma attached to the participants was not completely due to the disease itself. Embedded social and cultural values regarding ethnic minorities among Chinese Hong Kong citizens also played a considerable role in contributing to stigmatisation; indeed, experiencing stigmatisation had been common for all the participants since initially settling in Hong Kong. One participant recalled his experience upon first arriving in Hong Kong:

Many Hong Kong people were unfriendly to me when I first came here. They called me “black ghost”, and they kept a distance from me. They labelled us as AIDS carriers, and they were afraid of me. No matter when I went to a shop or a restaurant, I was not welcomed by them. Even now, still not many Hong Kong people can accept me. They still love calling me as “black ghost”. Also, every time when I take public transport, they will not sit or stand next to me. I still cannot understand why, but definitely I feel bad with it.

All the participants had worked in Chungking Mansions, and some of them recalled how this building was stigmatised by Chinese Hong Kong citizens because of the ethnicity of the people inside:

For almost 20 years ago, I was living and working in Chungking Mansions. At that time, no Hong Kong people would go inside [the building]. Although there was a shopping mall, I could hardly see any Hong Kong people coming in. Almost all the people in this building were Indians, Pakistani, and Africans. Many Hong Kong people were afraid of us, and they were afraid of this building, too. Maybe we are poor in their [Hong Kong people] eyes, or maybe our black skin is just too horrifying to them, or maybe they thought that we would rob them. “Black ghost” was the first Chinese word that I learnt here, and even up till now, some Hong Kong people still like calling us as “black ghosts”.

A few participants wanted to work for major companies in Hong Kong. However, their marginalised social position in the city often served as a barrier preventing them from working in the mainstream community. The participant with a university education recounted his unpleasant experience job searching when he came to Hong Kong:

I studied computer engineering in a university in my home country. With my university degree, I believe that it won’t be too difficult for me to find a job in information technology in Hong Kong, especially [because] there was an IT boom all over the world some years ago. I believe Hong Kong should be a good place that can offer me a good career prospect. However, I am just too naïve since what I have thought about is obviously my false hope. When I finished my first contract here, I had to find another job in order to extend my work visa and stay in Hong Kong. However, most Hong Kong IT firms were unwilling to hire me. I had been to many IT firms for job interviews, and many employers looked shocked when they saw me. No one hired me, but I had to make a living; so I worked in a restaurant at Chungking Mansions for some years. It was not until recently that an international company’s Hong Kong’s office gave me an offer for an IT position. Now I realise that not many Hong Kong people can accept Africans.

### How stigmatisation experiences shaped perceptions of EVD

The EVD-associated stigmatisation experienced by the participants both intensified the marginalisation caused by their ethnicity and shaped their perceptions of EVD. Most of the participants were unaware of the dangers of EVD before they experienced tangible stigmatisation in Hong Kong. Although the participants’ family members were living in Africa, some in countries with EVD cases, the participants commonly perceived the disease as too remote to affect them. However, the EVD-associated stigma that they experienced in Hong Kong shaped their perceptions of the disease as being shameful and horrifying. However, some of the participants adopted their own cultural values and beliefs to understand EVD, interpreting the disease as a form of retribution that had been introduced by Westerners.

#### A shameful disease

Because of the participants’ stigmatisation experiences, they perceived EVD as a shameful disease. As one participant stated:

I can imagine that if I get Ebola, I will be discriminated against further in Hong Kong. Many Hong Kong people are afraid of us now, thinking that we all have Ebola. If any of us really gets Ebola, it will be ashamed for us, because we really have Ebola and will surely be discriminated against further. If I really get Ebola, certainly I will not go to hospital; otherwise people will know and discriminate against me definitely.

The participants commonly perceived EVD as a disease of shame for all Africans. Because the disease originated in Africa and the 2014 outbreak occurred there, in addition to the stigmatisation from Chinese Hong Kong citizens, almost all the participants perceived the disease as being shameful for Africans. As one participant commented:

Ebola is a shame to me, and it is a shame to Africa. We are just too poor to get the disease. This disease does not hit in wealthy and Western countries, but it just hits Africa. Just like AIDS, it also hit in Africa at the very beginning. Why always Africa? It seems to me that it is our fate to suffer from these deadly diseases, because we are just too poor, and our hospitals are just too backward. It is a shame for me to tell others that I am from Africa, because other people will just think about poor, backward, and disease when they heard “Africa”.

#### A horrifying disease

Most of the participants were unaware about the dangers of EVD at the beginning, and had little knowledge about the disease. Their impressions of the disease were mainly shaped by the mass media and, most crucially, by the stigmatising behaviour that they experienced in Hong Kong. Because of their lack of knowledge of the disease and its high mortality rate, perceiving EVD as horrifying was a popular perception among the participants, though some of them believed that the mass media had been exaggerating the severity of EVD in depicting it as horrifying. As one participant commented:

I do not know exactly what Ebola is. I just know that many people are died of it, and there is no treatment for it. That is all I know from the news. Many Hong Kong people are scared of me and they keep a distance from me, so I guess the disease should be transmittable. Some Hong Kong people stare at me and mumbling “Ebola, Ebola”. Their reaction makes me feel that Ebola should be really very horrifying. However, when I ask my family members in Nigeria about Ebola, they do not know much about it. Ebola sounds horrifying in Hong Kong, but not in my home country.

The mysterious image of EVD also contributed to the participants perceiving the disease as horrifying. Almost all the participants were unable to receive much information about EVD. Their lack of information about the disease was mainly due to their limited social network for accessing information about the disease, which contributed to the mysterious image of EVD as a result. As this participant indicated:

I do not know what Ebola is, and do not know how one can get Ebola. The disease is just like a mystery, so it is even more horrifying to me. I do not know where I can obtain more information about Ebola. Although I live in Hong Kong, I do not have many friends here. I do not have television at my home so I cannot watch the news. I do not buy newspapers because English newspapers are expensive. I can just ask my African friends but none of them know much about Ebola, too. Probably the only source of information is from the Hong Kong customers, but they do not come to eat now. I have no way to get more information.

#### A disease of retribution

Because of the difficulties in obtaining reliable information about EVD, more than half of the participants used their own cultural beliefs and values to interpret the disease. One participant explained how he used his existing cultural belief to understand EVD:

In my country, we believe bad disease will come to find bad guys, so I think Ebola only infects bad guys. Bad guys behave badly, and they will annoy the god. When the god is annoyed, bad guys will be died of a painful disease as a punishment. Ebola is a punishment for bad guys. If you behave well and are a good guy, the god will not punish you and you will never get infected. But if you behave badly, you will die of this painful disease as punishment no matter how careful you are.

Such cultural beliefs affected if and how the participants took precautions against EVD:

Ebola is a punishment to bad guys, and they will bleed to death painfully. I am a good guy, and I never do bad things. I am honest to my customers and never cheat people, so I am not much afraid of this disease. I will not get infected because I am a good guy. If you are a good guy, then no matter how dirty you are, you will never get the punishment. If you are a bad guy, then no matter how clean you are, you will still get this painful disease, because it is a punishment for you.

#### A disease introduced by Westerners

Nearly half of the participants said they believed that EVD had been introduced by Westerners. These participants widely believed that EVD was imported from Western countries through the “invasion” of Westerners travelling to Africa for business. In many cases the participants often used their cultural beliefs to connect the Westerners with the disease and their own social stigmatisation. One participant expressed his hatred towards Westerners for, as he believed, bringing EVD to his home country:

Originally, my country is very beautiful, clean, and peaceful. We rarely got sick at all. However, after these Western people have come, we then get sick more often. It is them who bring along the bad spirits to us. These bad spirits make us sick. They buy all our lands and we have to work for them. We can no longer work in our own farms. They exploit our people to work for them. They cut down many trees as well, and this annoys the tree spirits. Because the tree spirits are annoyed, we get sick as a punishment. It is them who bring Ebola to us. It is their fault, but we have to suffer.

Interlocking with their cultural belief of EVD as retribution, some participants considered EVD as punishment of the Westerners for their bad behaviour and exploitation of the Africans:

The Western people come to different parts of Africa to exploit us, no matter [in terms of] work or health. They take many of our lands, and this annoys our god of land. We get sick more often, because the god is unhappy with their taking of our lands. Ebola is a punishment from the god, so it [the disease] is brought by the Western people. As we work for them, the god is annoyed with us, too, and so we also have to get the punishment. If it is not because of them, there will not be any Ebola. It is a punishment for them, but we have to suffer, because the god misunderstands us for helping them.

## Discussion

The interview data demonstrated the intertwining relationship between EVD-associated stigma and the embedded social and cultural values regarding ethnic minorities in Hong Kong. The stigmatisation experienced by the participants in their workplaces and the community was not merely induced by the EVD outbreak; rather, it was a continuation of cultural stereotypes of Africans among the Chinese Hong Kong citizens, and made the existing stigma more tangible. Such disease-associated stigmatisation of the participants played a considerable role in shaping their perceptions of EVD and their corresponding health behaviour.

Consistent with previous literature [4, 18], the participants, who occupied a marginalised social position in Hong Kong because of their ethnicity, were vulnerable to disease-associated stigmatisation during an epidemic. Marginalised social groups, such as migrants belonging to ethnic minorities, in particular Africans, are more vulnerable to being scapegoated as high risk groups for deadly communicable diseases [19, 20]. Some participants had been stigmatised as possibly having AIDS after first arriving in Hong Kong, and they were stigmatised as possibly having EVD during the EVD outbreak.

In Hong Kong, ethnic minorities are often marginalised; they often encounter difficulties in study, work, and daily life [21]. Government statistics also shows that a high proportion of Chinese Hong Kong citizens reported unpleasant interactions with ethnic minorities [22]. Even people belonging to ethnic minorities who are born in Hong Kong are often not perceived as “Hong Kong citizens” [23]. Because of the language barrier, these people often have to go to government-designated schools for ethnic minorities [24], thus their marginalised social position and social isolation from the main Hong Kong community are created and reinforced from childhood. When they grow up, they often encounter substantial difficulties in finding employment, even though they may have a university education [23]. The situation may be even worse for Africans; in a ranking of Chinese Hong Kong citizens’ acceptance of ethnic minorities, Africans placed in the bottom third [22]. The participants of the current study, who were not born in Hong Kong, were expected to encounter even more hardship in Hong Kong. As indicated, the participants were often labelled “black ghosts” and prevented from obtaining their career gaols. Many of the participants were unable to gain employment in major companies, forced to work instead in communities mostly comprising ethnic minorities. This further contributed to their social isolation and marginalised social position. They had very limited interaction with Chinese Hong Kong citizens in both their workplaces and in the community. Therefore, the participants were excluded from the main community and could interact only within their own social network with their own and/or other ethnic minorities who encountered the similar situations. Such social isolation and marginalisation made obtaining reliable information about EVD difficult for the participants, rendering them more vulnerable to being infected by not merely EVD but also other communicable diseases.

The participants’ marginalised social position might also have served as a barrier to them seeking health care. It is not uncommon for ethnic minorities to encounter unpleasant experiences when interacting with health care providers in Hong Kong [25]. Because of their marginalised social position, they receive little health care support [25]. These unpleasant experiences in seeking medical treatment can undermine ethnic minorities’ confidence and trust in Hong Kong’s health care providers [25], which may result in unwillingness to seek treatment. This serves as a potential risk for Hong Kong’s public health, especially because some participants came into contact with family members living in countries with EVD cases.

The health care providers’ adoption of infection control measures also embarrassed the participants. Wearing full protective gear was advised by the health authorities for only when dealing with suspected EVD patients, with standard precautions considered sufficient in general outpatient settings [26]. However, this official advice was not followed, according to the participants’ recounting of receiving follow-up treatment for chronic conditions in public hospitals. The different protective gear used by the health care staff when dealing Hong Kong patients and the participants revealed the health care providers’ cultural perceptions and stereotypes of the Africans; specifically, the Africans were widely stereotyped as a high-risk group and “dirty” patients. Such different treatment by health care providers of the participants was not exceptional; health care providers were already noted as lacking sensitivity in providing treatment to marginalised and stigmatised social groups [27]. Stigma and marginalisation of certain social groups can interfere with health care providers’ decisions and behaviours towards marginalised patients [27]. Because of the ethnicity of the participants, health care providers behaved differently when providing treatment to them (as demonstrated by their wearing of protective gear). This different treatment in medical encounters and the stereotyping by health care providers both intensified the participants’ unpleasant experiences with health care providers and deepened their sense marginalisation and stigmatisation in Hong Kong during the EVD outbreak.

Because Africa was the epicentre of EVD, it was perceived as a “dirty” place by most Chinese Hong Kong citizens following the EVD outbreak. The participants, hence, were discouraged from returning to their home countries during the EVD outbreak, and could have faced serious consequences from the community if they did. Feelings of insecurity towards marginalised social groups was noted to be common among ethnic majorities [28], because the social practices and cultural values of the marginalised groups clash with the existing social and cultural values of the community [28]. During epidemics, such insecurity towards ethnic minorities can be heightened. To overcome the sense of insecurity and ensure social stability, marginalised social groups are often oppressed [28]. The study participants were oppressed by being pressured to avoid connections with the “dirty” zones during the outbreak to ensure that their practices and behaviours were aligned with those of the ethnic majorities. A participant returning to his home country was perceived as an act against the majorities’ social values. As a result, members of the ethnic majorities monitored the participants to ensure that they had any contact with a “dirty” zone.

Even among different ethnic groups stigmatised each other, according to the participants. Indeed, the literature shows that it is common for ethnic groups in the same community to come into conflict with one another in attempts to protect their own rights [28]. The participants’ experiences were no exception. Government statistics show that pleasant interactions with other ethnic groups is low among non-Chinese people in Hong Kong [22]. Because making a living is often difficult for most ethnic minorities in Hong Kong, the participants were perceived as competitors and intruders by the other ethnic groups. The marginalised positions of ethnic groups in Hong Kong made the competition in their shared job market even fiercer. In addition, because of the marginalisation of different ethnic minorities in Hong Kong, the intent of members of non-African minorities to distinguish themselves and their ethnic group from other members of other ethnic groups (such as the participants) was stronger, with the aim of avoiding further stigmatisation. Distinguishing themselves from the “dirty” participants became a survival strategy for other ethnic minorities in Hong Kong during the EVD outbreak.

The EVD-associated stigmatisation in Hong Kong shaped the participants’ perceptions of the disease. Because of their social seclusion, the participants experienced substantial difficulty in accessing reliable information about EVD. Their lack of knowledge of EVD led them to hold a mysterious and horrifying impression of the disease, resulting in a sense of insecurity. To cope with this insecurity, the participants made sense of the disease through their cultural beliefs. Africa’s historical background, particularly its colonial history, had created unpleasant social and cultural feelings in the participants. The new social system and cultural values introduced by Westerners clashed with the original environment, social systems, and cultural values. These social and cultural experiences led the participants to associate EVD with the unpleasant history and Westerners, and they thus perceived EVD as a disease of retribution introduced by Westerners.

The attitudes and behaviours of the ethnic majorities in Hong Kong during the EVD outbreak also shaped the participants’ perceptions of EVD, leading them to view it as a shameful and horrifying disease; EVD was not merely perceived as deadly—it also conveyed another stigma to the participants. Both the colonial history of Africa and the double burden of stigmas, in relation to ethnicity and disease, that the participants had experienced in Hong Kong greatly shaped their perceptions of EVD.

The participants’ perceptions of EVD influenced their corresponding health behaviour, which could have impaired the effective control of EVD. The participants perceived EVD as a shameful disease. As noted in earlier studies, a disease-associated stigma can motivate patients to conceal health conditions and delay seeking treatment [12]; the disadvantaged social position of marginalised populations can even influence how they access health services when seeking treatment [19]. Thus, the stigma-attaching nature of EVD might have prevented the participants from seeking timely treatment if they had contracted the disease.

Cultural beliefs could also have served as a barrier for the effective control of EVD in the participants. The participants’ cultural beliefs, which interpreted EVD not as a highly communicable disease but as a retribution for immoral behaviour and a disease introduced to Africa by Westerners, demotivated them to actively guard against the disease. Moreover, the participants’ distrust and blame of Westerners could have prevented them from receiving Western public health or medical assistance. To the participants, the Westerners were evil “intruders” who had gone to Africa to take the resources there, and who had brought various diseases in doing so. Such cultural beliefs served as a remarkable obstacle to the participants trusting biomedicine, which would have made EVD difficult to control among them; this situation played out in reality in the infected countries in Africa. In addition to the participants’ marginalised social position and the stigma-attaching nature of the disease, the participants could easily have become “hidden patients” had they been infected by EVD. Some of the participants perceived the dangers of EVD as having been exaggerated by the mass media. This might have further reduced their motivation to take preventive measures against the disease.

The experiences of the participants demonstrated the extreme health inequality between the ethnic minorities and ethnic majorities in Hong Kong caused by the minorities’ marginalised social position and narrow social network. Such health inequality resulted in many “blind spots” in health education and health care among ethnic minorities in Hong Kong, and this further marginalised these people, rendering them highly vulnerable to and at high risk from EVD. Although Hong Kong’s health authorities had disseminated health education pamphlets about EVD prevention to African communities in the city [29], these had failed to reach the participants. Because there are other ethnic minorities (e.g., Indians, Nepalese, Indonesians, and Thais) in Hong Kong, and because the social positions of these ethnic minorities are similar to those of the participants, these other ethnic minorities may be assumed to also have encountered difficulty in accessing reliable infection prevention information [30]. Without reliable health information, the participants’ cultural beliefs and social experiences were the predominant determinants of their perceptions of and ultimately health behaviour in response to EVD. Previous studies have noted that health inequality can results in poor and marginalised people being more vulnerable to various infectious diseases [31]. Hence, ethnic minorities require more socially and culturally responsive health promotion and education to provide them with accurate information about EVD and preventive measures against the disease.

### Limitations

This study recruited only Africans who had obtained Hong Kong residency. Thus, this study was unable to investigate the experiences of migrant Africans with a shorter stay in Hong Kong. Because of the communication issue, sampling bias might occur since only the participants who are able to communicate in English were sampled. Hence, the findings might reflect the Africans who had a higher education level. In addition, other ethnic minorities were excluded from the sampling, which made understanding other ethnic groups’ experiences more difficult.

## Conclusions

This study investigated the stigmatisation experiences of Africans residing in Hong Kong during the 2014 West Africa EVD outbreak. Their stigmatisation was not solely due to EVD; rather, it was a continuation of the embedded social and cultural values towards ethnic minorities in Hong Kong. EVD was a trigger that intensified the existing stigma and made it more tangible for Africans living in Hong Kong. The experiences of being stigmatised shaped the participants’ perceptions of EVD. In addition, because of the participants’ marginalised social position and isolation from the main community, they had limited access to reliable information about EVD. As a result, they used their own cultural beliefs to understand EVD, which might ultimately have influenced their health behaviour towards the disease. The experiences of the participants showed that ethnic minorities in Hong Kong were in need of more culturally responsive social and health care support to enable them to obtain reliable information about EVD and preventive measures against it.
